# Microglial Pruning: Relevance for Synaptic Dysfunction in Multiple Sclerosis and Related Experimental Models

**DOI:** 10.3390/cells10030686

**Published:** 2021-03-20

**Authors:** Maria Concetta Geloso, Nadia D’Ambrosi

**Affiliations:** 1 Department of Neuroscience, Section of Human Anatomy, Università Cattolica del Sacro Cuore, Largo Francesco Vito 1, 00168 Rome, Italy; 2 Department of Biology, University of Rome Tor Vergata, Via della Ricerca Scientifica 1, 00133 Rome, Italy; nadia.dambrosi@uniroma2.it

**Keywords:** microglia, pruning, neurodegeneration, multiple sclerosis, experimental autoimmune encephalomyelitis (EAE), synaptic loss

## Abstract

Microglia, besides being able to react rapidly to a wide range of environmental changes, are also involved in shaping neuronal wiring. Indeed, they actively participate in the modulation of neuronal function by regulating the elimination (or “pruning”) of weaker synapses in both physiologic and pathologic processes. Mounting evidence supports their crucial role in early synaptic loss, which is emerging as a hallmark of several neurodegenerative diseases, including multiple sclerosis (MS) and its preclinical models. MS is an inflammatory, immune-mediated pathology of the white matter in which demyelinating lesions may cause secondary neuronal death. Nevertheless, primitive grey matter (GM) damage is emerging as an important contributor to patients’ long-term disability, since it has been associated with early and progressive cognitive decline (CD), which seriously worsens the quality of life of MS patients. Widespread synapse loss even in the absence of demyelination, axon degeneration and neuronal death has been demonstrated in different GM structures, thus raising the possibility that synaptic dysfunction could be an early and possibly independent event in the neurodegenerative process associated with MS. This review provides an overview of microglial-dependent synapse elimination in the neuroinflammatory process that underlies MS and its experimental models.

## 1. Introduction

Multiple sclerosis (MS) is a demyelinating, inflammatory, immune-mediated central nervous system (CNS) pathology in which neuronal death has traditionally been considered the consequence of prolonged and severe axonal damage related to myelin loss. Nevertheless, primitive neurodegeneration independent of demyelination, involving different grey matter (GM) regions of the CNS [1,2,3,4], is emerging as an important contributor to patients’ long-term disability [1,5,6]. Primitive GM damage is believed to play a primary role in the pathogenesis of the complex behavioural, psychiatric and cognitive disturbances that, together with sensory-motor impairment, affect the employment potential, social activities and quality of life of MS patients [1,5]. Among these disturbances, cognitive decline (CD) is now recognized as a significant clinical feature of MS, occurring in up to 70% of patients at some time in their disease course [7] and worsening over time, independently of disease stages and clinical phenotypes [1]. However, both comprehensive knowledge of the neurobiology of this phenomenon and effective therapeutic approaches aimed at reducing its progression are still limited [8]. 

Numerous studies have attempted to clarify the neurobiology of primitive neuronal death in MS, and a large body of evidence points to the key role of synaptic dysfunction as an early pathogenic event occurring even before overt neuronal damage [1,5]. This is particularly relevant, since impairment of the synaptic compartment is emerging as the first manifestation of neuronal damage in a wide range of neurodegenerative diseases, including Alzheimer’s disease (AD), Parkinson’s disease (PD), Huntington’s disease (HD), amyotrophic lateral sclerosis (ALS) and spinal muscular atrophy (SMA) (reviewed in [9,10,11]), suggesting the possibility of common mechanisms underlying these pathologies.

Certain intrinsic properties of the synapse may be responsible for its selective vulnerability through cell-autonomous mechanisms [12]. Indeed, because of its highly specific role in neurotransmission, the synaptic compartment is equipped with subcellular components that allow it a certain degree of autonomy from the cell body [13,14]. For instance, the synapse is characterized by specific subtypes of mitochondria required to meet the high-energy demand of this structure [15]. Consequently, altered mitochondrial distribution at the synapse as a result of impaired axonal transport is believed to play a significant pathogenic role in synaptic damage associated with ALS and hereditary spastic paraplegia [16]. Similarly, alterations in mitochondrial dynamics are thought to play a prominent role in other neurodegenerative diseases marked by early signs of synaptic impairment, such as AD, PD, HD, ALS-frontotemporal dementia, SMA and peripheral neuropathies [9,17]. Highly specialized domains, such as the pre- and post-synaptic compartments, are also a peculiar feature of synapses [18]. Impairment of the complex molecular machinery at this level has been associated with early synaptic dysfunction followed by structural loss [9], as exemplified by mutations in the amyloid precursor protein, a component of the presynaptic active zone involved in familiar AD [19], or in the presynaptic Leucine-rich repeat kinase 2 responsible for autosomal dominant, late-onset PD [9,12,18,20].

In addition to the above, growing evidence supports the notion that dysfunctions in neuron–glia signalling contribute to synapse loss through non-cell-autonomous mechanisms, as reported in neurodegenerative and neurodevelopmental disorders [21,22]. Indeed, both astrocytes and microglia contribute to the physiology of the synaptic contact [21,23]. Astrocytes are known to establish close contact with pre- and post-synaptic elements to form the so-called “tripartite synapse”. This represents the structural basis for a wide range of dynamic interactions, including the clearance of neurotransmitters and the exchange of signalling molecules that control synapse formation, maturation and elimination, eventually resulting in a tight regulation of synaptic plasticity [21,23]. Microglia, besides being able to rapidly react to a wide range of pathologic and/or homeostatic changes in the brain acting as a “sensor” for pathologic events [24], are deeply involved in shaping neuronal wiring [24]. They are able to react rapidly to neuronal activity by modifying their motility properties and physical interactions with synapses [25]. Through their highly plastic processes, microglia are in close contact with boutons, spines, synaptic cleft and peri-synaptic astrocytes, thus forming the “quadripartite synapse”, in which the various cytokines and growth factors produced by these cells actively participate in synapse functioning and remodelling [1]. They provide an additional contribution to the fine-tuning of neuronal circuits through the activity-dependent elimination of weaker synapses by phagocytosis [25,26]. Notably, pathologic activation of this phenomenon (aberrant pruning) is emerging as one of the mechanisms principally involved in early synaptic loss during different neurodegenerative processes [27]. As immune molecules play a central role in microglia-mediated synapse elimination [28] and many of these pathways also play relevant roles in the immune-mediated pathogenesis of MS, the present review aims to revisit the role of alterations to the dynamic microglia–synapse interactions in the etiopathogenesis of the synaptopathy characteristic of the early phases of GM damage in MS, with a special focus on the mechanisms leading to aberrant regulation of microglia-mediated synapse elimination.

## 2. Overview of Microglia and Synaptic Pruning

Among the cells composing the CNS, microglia are considered the resident macrophages, as one of their core functions is to promote the phagocytic clearance of pathogens, apoptotic cells and tissue debris in order to maintain local homeostasis, resolve inflammation and support tissue repair [29]. Phagocytosis is a complex process that involves the recognition, engulfment and digestion of unwanted material. To accomplish these tasks, microglia first recognize “find me” signals, then migrate toward their source and, on detecting “eat me” signals, initiate phagocytosis. The first stage of this process consists in cytoskeletal rearrangements that lead to the formation of phagocytic cups, followed by the engulfment and degradation of internalized material by the system of endosomes and lysosomes. As specialized phagocytes, microglial cells are endowed not only with immune receptors to detect pathogens or tissue-damage-associated molecular patterns, but also with an array of receptors necessary to mediate all the steps of phagocytosis [30]. They also express different receptor types to respond to microenvironmental signals such as chemokines, cytokines and immunoglobulins, which in turn stimulate the production by microglia of a variety of effector molecules, often generating a self-sustaining pro-inflammatory reaction [31]. Microglia also use their immune-phagocytic functions to shape and remodel synapses in various circumstances [32].

The removal of excessive or unnecessary synaptic networks is a central event in promoting the refinement and maturation of the developing CNS [25]. Synaptic pruning also takes place in the peripheral nervous system, where Schwann cells in particular are involved in the maintenance and strengthening of the neuromuscular junction [33]. Microglia directly contact and affect multiple synaptic elements in a very dynamic manner, and during the first weeks after birth they engulf pre- and/or post-synaptic elements, as demonstrated in the retinogeniculate system, hippocampus, cerebellum and cerebral cortex [34]. The identification of long-lasting synapses is related to the amount and timing of neural activity, both of which are parameters that are critical in determining which synapses are retained and which are instead marked for removal. By sensing local neurotransmitter release, microglia are able to discriminate between active and weak synapses, engulfing only those displaying low activity [25]. Microglial engulfment of synapses likely varies in different developmental stages, brain regions and disease states. The timing of synapse pruning varies according to the brain area: in human primary visual cortex pruning is completed between the 4th and 6th years of life, while in areas involved in complex cognitive functions, such as the prefrontal cortex, synaptic pruning often continues until the end of adolescence [35]. The particular system through which unwanted synapses are identified also varies within the brain. An example of this is the complement system, which is expressed by both neurons and glia and whose function is not only to protect the nervous system from infection and inflammation, but also to identify unwanted synapses in the post-natal developing brain and in brain diseases [36]. Its use in developmental synaptic pruning occurs especially in the retinogeniculate system [34]. In adult life, however, pathways using the complement cascade may have a wider role in establishing appropriate connectivity among neurons [34].

While a description of synaptic pruning in the developing CNS lies far beyond the scope of this review and has been eminently accomplished elsewhere, we are concerned here with CNS prototypical pruning paradigms, such as those occurring in the retinogeniculate circuit of the visual system and in the hippocampus [29,34]. Proteins belonging to the innate immune system, such as those of the complement cascade C1q and its downstream molecule C3, are tags employed to mark synapses in the visual thalamus in order to elicit their phagocytosis by microglia expressing complement 3 receptor (C3R). Neuronal C1q is normally upregulated during development to identify unwanted synapses, and behaves as an “eat me” signal [37,38]. Synaptic refinement occurring during visual plasticity also requires the involvement of P2Y12R purinergic signalling in the targeting of microglial processes toward synaptic elements requiring elimination [39]. In the developing hippocampus, different mechanisms of synapse elimination functioning in a complement pathway-independent manner have also been identified. During post-natal development, microglia affect synapses through activation of the CX3CR1 receptor by its ligand, fractalkine, produced by neurons. Released fractalkine recruits microglia, thus behaving as a “find me/eat me” signal and determining the removal of excessive synaptic connections. Accordingly, CX3CR1 knockout mice display impaired synaptic pruning and disturbed social behaviour [40].

**Figure 1:**
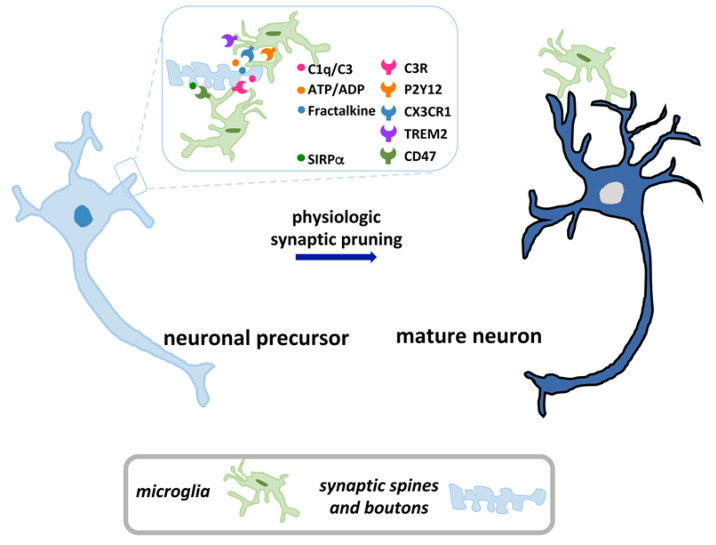
Physiologic synaptic pruning. During development, microglia remove excess synapses from neuronal precursors via diverse ligand-receptor mechanisms, thereby contributing to neuronal maturation.

A different pathway is mediated by the triggering receptor expressed on myeloid cells 2 (TREM2) and its signalling adaptor DAP12, which is responsible for promoting the phagocytosis of synaptic proteins during periods of hippocampal circuitry refinement, particularly in the CA1 area [41]. Indeed, *Trem2^−/−^* mice show defective microglial activation, increased synaptic density and impaired connectivity [41]. All these mechanisms are summarized in Figure 1.

With regard to the mechanism of engulfment, fluorescence microscopy and correlative light and electron microscopy studies have recently shown that only presynaptic boutons are digested by microglia in organotypic hippocampal slices, while, at post-synaptic sites, the contact with microglia leads to the remodelling of dendritic spines, as attested by the formation of transient filopodia [42]. The removal of presynaptic elements occurs through a special type of phagocytosis, called trogocytosis, involving the transfer of plasma membrane fragments to microglia without the formation of a phagocytic cup [42].

While different systems for the identification of supernumerary synapses have been recognized, it is still not clear how normal synapses are instead spared removal. It is believed that a class of molecules, which are responsible for preservation and strengthening [43] and known as “don’t eat me” signals, may protect synapses from damage during pruning. As an example, in the developing retinogeniculate system the interaction between CD47, which is present on neuronal membranes, and its receptor, SIRPα, which is localized on microglia, inhibits synaptic phagocytosis, thus behaving as a “spare me” signal [44] (Figure 1).

Together with microglia, astrocytes are also involved in modulating synaptic activity at different levels, being implicated in synapse formation and elimination and in neuronal plasticity, therefore functioning both during development and to refine adult circuitries [21]. Although they can directly mediate synapse elimination via different pathways, including the activation of MEGF10 and MERTK, astrocytes participate in synapse removal indirectly, through the secretion of transforming growth factor-β and the consequent increased deposition of C1q on developing neurons, which ultimately activates microglial phagocytosis [23].

Disturbances in normal pruning mechanisms that occur during development could lead to faulty wiring and contribute to altered neuronal circuits. A proper balance between synapse formation and elimination (pruning vs. maintenance) is clearly necessary to preserve homeostasis between excitatory and inhibitory synapses, so its dysregulation could account for neuropsychiatric diseases such as schizophrenia and autism spectrum disorders [45,46]. The correlation between aberrant pruning and CD is well exemplified in Nasu–Hakola disease, an autosomal recessive disorder involving loss-of-function mutations in the phagocytic *TREM2* gene and characterized by progressive presenile frontotemporal dementia [47].

In addition to situations where dysregulated pruning likely occurs at developmental stages, many recent data point to a reactivation of the pruning machinery in adults as a consequence of the decrease in synaptic activity that occurs during ageing or as a result of neuronal damage [48]. The loss of presynaptic terminals and dendritic spines, together with the activation of glial cells, are early pathogenic mechanisms that strongly correlate with CD in a number of neurodegenerative diseases. Microglia play a major role in synapse elimination in such conditions. For instance, synapses are recognized as vulnerable sites after the local accumulation of aggregates of misfolded proteins, a process that is typical of many neurodegenerative disorders. Intrinsic genetic defects in glia are also possible causes of aberrant synaptic pruning, determining a dysfunctional regulation or incorrect reactivation of refinement pathways [11].

Regardless of the initial trigger, the reactivation of aberrant pruning can cause synaptic loss as a consequence of excessive complement deposition. Studies concerning this aspect of neurodegeneration have been performed in mouse models, but observations consistent with this notion have also been reported in post-mortem examinations of human brain samples [49]. In this instance, AD represents a form of neurodegeneration in which aberrant pruning and exacerbated neuroinflammation involving activated microglia are strongly implicated in the genetics and neuropathology of the disease. Many AD risk genes, including *APOE*, *CLU*/*apoJ* and *TREM2,* are expressed or enriched more in microglia than in other types of brain cell [50]. Interestingly, AD-associated genes include *CR1*, and it has been widely shown that the C1q-C3 complement system, which has a low baseline expression in adults, is instead strongly upregulated and involved in synapse loss in AD [51]. It is likely that Aβ oligomers and tau aggregates are inducers of complement cascade reactivation, suggesting that their toxicity is also determined through the pathogenic mechanism of microglia-mediated synaptopathy [52,53].

Although less well documented than in AD, evidence for a role of microglia in early synaptic dysfunctions, synapse engulfment and alterations in excitatory/inhibitory circuits has also been found in other neurodegenerative diseases, such as PD and ALS. All these features suggest that microglial pruning can contribute to the CD associated with these disorders [11].

## 3. Microglia and Synaptopathy in MS and Experimental Autoimmune Encephalomyelitis (EAE)

### 3.1. Schematic Timeline of MS Pathology

It is well established that the immune system provides a direct contribution to the myelin loss and neuronal damage that characterize MS through antigen-specific targeting of myelin and other components of nervous tissue [54]. The breakdown of the blood–brain barrier as a consequence of still unidentified causes allows increased rolling, adhesion and diapedesis of immune cells within the CNS, culminating in an invasion of the CNS by T-cells. This event is followed by the recruitment and activation of other inflammatory cells, such as macrophages, astroglia and microglia, which in turn release cytokines and other mediators, contributing to injury to myelin and neurons. It is believed that additional mechanisms, such as demyelinating antibodies and complement factors, are also required to induce demyelinating plaques (reviewed in [55]).

The sequence of events during the formation of MS lesions is well established [54,56,57]. The earliest stage is represented by “preactive lesions”, corresponding to early changes in normal-appearing WM and GM regions [56]. Their main feature comprises nodules of activated microglia, associated with scattered CD45-positive lymphocytes, typically in the absence of demyelination [56]. Notably, while microglia still maintain their ramified morphology [56], astrocytes already show a hypertrophic phenotype in this phase [58]. These early lesions may be reversible, but usually evolve towards the acute plaque, characterized by discrete areas of myelin loss and perivascular cuffs of inflammatory cells (for review see [54]). Foaming macrophages and markedly activated microglia are diffusely present throughout acute plaques [57], accompanied by reactive astrocytes (for review see [54]). In particular, in this phase, active microglia induce astrocytes to acquire a neurotoxic phenotype [59], thus increasing their contribution to the severity of tissue damage. The next stage is the chronic plaque, characterized by reduced inflammatory infiltrating cells, extensive myelin loss and severe axonal damage [54,57,60]. In the final stage, a hypocellular centre forms, myelin is lost completely, inflammation is significantly reduced, and hypertrophic astrocytes form a scar [56].

Inflammatory and demyelinating lesions are also diffusely present in the GM of MS patients [54], involving both the cerebral cortex and the hippocampus, as well as grey subcortical nuclei, including the thalamus, basal ganglia, hypothalamus, cerebellum and spinal cord [61]. At the pathologic level, GM lesions are characterized by less pronounced inflammatory features, with reduced infiltrating lymphocytes and less severe blood–brain barrier disruption, while a loss of neurons, glia and synapses is a common feature [54]. Notably, post-mortem studies have shown that neuronal loss is also detectable in normal-appearing GM, suggesting that neuronal damage in MS may also occur independently of GM demyelination [6].

### 3.2. Overview of Synapse Loss in GM Damage Associated with MS and EAE

As in other neurodegenerative pathologies, synaptic dysfunction and loss of synaptic contacts among neurons, or “synaptopathy”, is a feature of GM neurodegeneration in both MS and its preclinical models, as extensively reviewed by other groups (for review see [5,28,62,63,64]).

**Figure 2:**
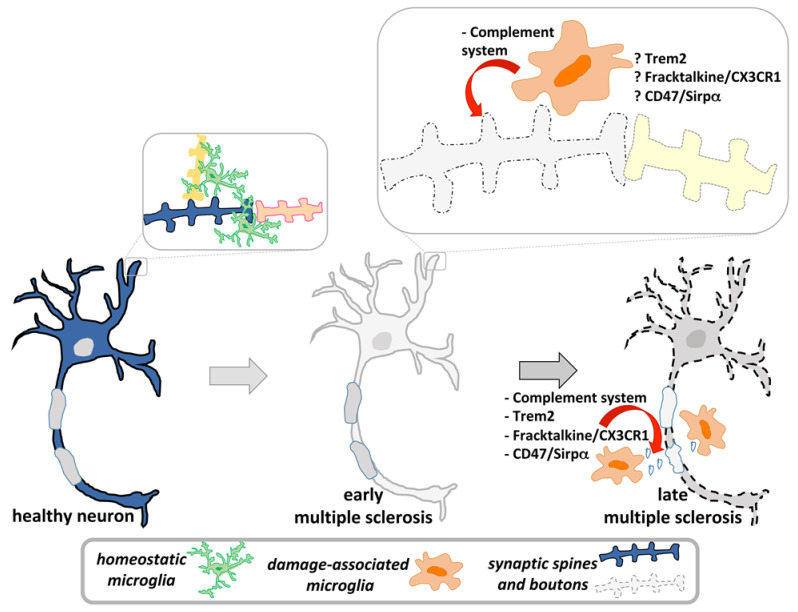
Synapse loss is an early event that characterizes primitive neuronal damage in multiple sclerosis (MS). Aberrant pruning, in which activated microglia play an important role, mediates this process, mainly through the complement pathway. With the progression of demyelination, the neuropathologic hallmark of the disease, microglial phagocytic activity is mostly aimed at clearing myelin debris.

Widespread structural loss of spines in non-affected cortical regions in MS [65], and similarly in EAE [63,66], appears as a primary event, occurring early in the course of the disease, independently of demyelination and axonal damage [2,3,65,67,68,69] (Figure 2).

At the molecular level, many findings point to the occurrence of reduced synaptic density and/or alterations in the expression pattern of pre-and post-synaptic proteins, which likely reflect impaired synaptic homeostasis [1,5]. Of special interest are findings concerning cortical regions functionally involved in cognitive processes, such as the hippocampus [67,70,71,72] and the prefrontal cortex [73,74], which further support the notion that early synaptopathy represents a central event for CD during MS progression [28]. In the non-demyelinated hippocampus of MS patients, in particular, Dutta et al. reported a reduced expression of, among others, synaptic proteins relevant for cognitive processes, such as calmodulin-associated serine/threonine kinase, the presynaptic adhesion molecules neurexins (NRXNs), and their post-synaptic ligands, neuroligins [67]. Notably, mutations in genes encoding for these proteins are associated with neurodevelopmental pathologies showing cognitive impairment [75,76,77]. Interestingly, results in the EAE experimental model essentially match those found in humans [70,71,72]. In addition, in the prefrontal cortex, the inflammatory changes brought about by the disease impact molecular mechanisms responsible for the specificity of synaptic connectivity, such as the alternative splicing of genes encoding for synaptic proteins, e.g., *NRXNS 1–3* [74]. It is known that NRXNs 1–3 splice variants selectively bind specific ligands in the post-synaptic compartment, thus fine-tuning the functional properties of the synapse [75,76]. Also of interest is the fact that altered splicing of the AS4 exon in these genes, found in EAE [74], has been directly linked to cognitive functions and memory formation [78,79].

In addition, the existence of sexual dimorphism in the variations in postsynaptic scaffolding protein expression has also been recently reported in EAE [80].

Changes in the arrangement of the molecular machinery of synaptic terminals is associated with the concomitant failure of synthesis, release, degradation and reuptake of neurotransmitters, culminating in disturbances in neural transmission. Excessive glutamate release and reduced GABAergic transmission, both of which lead to alterations in the excitatory/inhibitory balance and excitotoxicity, have been widely reported in MS and EAE (for review see [1,64,81,82,83,84]). Experiments in preclinical models clearly show that excessive glutamate transmission is an early event and that it is sustained by altered expression and phosphorylation of AMPA receptors [64]. Furthermore, recent findings indicate that “maladaptive” cortical hyperactivity can also be present in the remitting phase of the disease [85]. Excessive activation of glutamate receptors is known to cause excitotoxic neuronal damage by inducing the impairment of calcium buffering, the generation of free radicals and mitochondrial dysfunction [86]. Other neurotransmitter systems also show functional changes during MS. Reduced serotonergic, dopaminergic and cholinergic signalling have been described (for review see [1,5,83]), and together are considered an important pathogenic event underlying both CD and neurobehavioural changes in MS [84].

### 3.3. Role of Aberrant Microglial Pruning in Synaptic Elimination in MS and EAE

Persistent microglial activation involving WM and GM regions is widely documented in both MS and its preclinical models (for review see [87,88,89,90]).

Microglial nodules represent the hallmark of early preactive MS lesions [56], where they lose their homeostatic phenotype to acquire pro-inflammatory features, with consequent increased expression of signalling molecules involved in phagocytosis, oxidative stress, antigen presentation and T-cell co-stimulation [91]. A pro-active role in initiating the demyelination process has also been suggested [92,93]. Furthermore, imaging techniques for visualizing and measuring neuroinflammation in vivo have confirmed the presence of activated microglia also in normal-appearing WM and GM regions of patients [94]. Active microglia are diffusely present in active plaques, in which analysis of TMEM119, which is expressed by microglia but not by macrophages, showed that about 45% of macrophage-like cells were derived from resident microglia [91].

At the GM level, over-activated microglia are believed to play a crucial role in the pathogenesis of MS-related neuronal injury [6]. One of the mechanisms through which microglia provide their contribution to GM damage could be the impairment of astrocytic glutamate reuptake [95], thus increasing excitotoxicity [95].

Consistently, in EAE, high-dimensional single-cell mass and fluorescence cytometry indicate that the entire microglial population shows a homogeneously highly reactive profile, with a shift from a homeostatic to a damage-associated phenotype [96]. Activated microglia dominate the tissue reaction surrounding demyelinating lesions compared with blood-derived macrophages, as shown in the lysophosphatidylcholine-induced demyelination model [97].

Activated microglia also affect MS and EAE progression, exacerbating local inflammation, as highlighted by studies based on microglial inhibition or depletion [98,99], although the possibility that they may also exert protective roles, for instance through the modulation of CD4^+^ T-cells in the brain, has recently been proposed [100].

It is therefore not surprising that many findings indicate that activated microglia play a relevant role in MS-related synaptopathy. Indeed, a correlation between reduced cortical synaptic density and activated microglia has been described both in human samples [95] and in EAE [101]. In the latter model, the close proximity between microglial processes and axons has led to suggestions that early and persistent microglial activation, occurring independently of local T-cell infiltration, could be responsible for synaptic stripping [101,102].

In response to MS-induced inflammatory stimuli, microglia significantly affect the structure and function of synapses through different mechanisms. One such mechanism is the release of cytokines, known to modulate neuronal activity and synaptic plasticity in both physiologic and pathologic conditions [103], as extensively reviewed by other groups [5,82,104], the discussion of which is outside the scope of the present review.

Another mechanism is represented by the aberrant activation of the same molecular pathways that drive synaptic pruning during development, leading to synaptic engulfment and excessive synapse elimination, as shown by a growing body of evidence. Indeed, it has recently been shown that phagocytes act as “executioners” in the synapse loss that accompanies MS and experimental models of the disease, and that they select vulnerable synapses on the basis of their abnormal accumulation of calcium, which may function as an “attack me” signal [4]. In this regard, Krasemann et al. (2017) highlighted the occurrence of a homogenous transcriptional signature of an EAE-damage-associated microglial phenotype characterized by activation of a TREM2- apolipoprotein (APO)E-dependent program linked to phagocytosis and lipid metabolism [105]. Notably, the same phenotype also occurs in other neurodegenerative diseases, namely AD and ALS [87,105], both of which are characterized by early synapse loss [106,107]. In this regard, it is worth mentioning that recent findings highlight a role of APOE isoforms in the modulation of glia-dependent synaptic pruning, as well as in controlling the rate of accumulation of complement C1q at the synapse. These findings led to the hypothesis that altered activity of this group of proteins may increase synapse vulnerability in ageing and in AD [108]. Interestingly, the importance of the role exerted by TREM2 in aberrant synaptic pruning is now emerging, as shown in the APP/PS1 mouse model of AD, in which *Trem2* deletion improves synaptic loss in the early phases of the disease [109].

In MS and its preclinical models, TREM2 counteracts the progression of demyelination by clearing myelin debris and also affects the clinical course of the disease by modulating inflammation. In MS, increased levels of soluble forms of TREM2 are detectable in the cerebrospinal fluid (CSF) [110,111,112,113]. At the neuropathologic level, active demyelinating lesions show increased expression of TREM2 in myelin-laden phagocytes [114]. In the chronic progressive model of EAE, microglia show an upregulation of TREM2 starting from the early phases of the disease both in the spinal cord and in the brain. Notably, both the course of the disease and brain inflammation are exacerbated when TREM2 expression is blockaded [115]. Furthermore, in the cuprizone-induced model of demyelination *Trem2^−/−^* mice have been shown to exhibit an accumulation of myelin debris, increased axonal dystrophy and persistent demyelination [116,117]. Interestingly, recent findings in the same model showed that treatment with TREM2-agonistic antibodies enhances microglial myelin debris clearance and improves oligodendrocyte precursor cell differentiation [114].

Together with TREM2, other molecules involved in leading the phagocyte toward its target are believed to play a role in the microglial phagocytic activity promoted by MS, essentially aimed at clearing myelin debris. In particular, proper functioning of the fractalkine/CX3CR1 signalling pathway is required to manage tissue reaction to inflammation and demyelination (for review see [118]). Indeed, *Cx3cr1^−/−^* mice show increased levels of proinflammatory molecules and reduced levels of anti-inflammatory cytokines, associated with a more severe clinical course of EAE [119]. The influence of fractalkine/CX3CR1 signalling in the clinical course of the disease is highlighted by the observation that the occurrence of polymorphisms in the *CX3CR1* locus, which may cause defective binding to fractalkine, aggravates the progression of the disease [120]. Interestingly, impaired signalling between fractalkine and its receptor has been shown to be potentially relevant in neuronal damage accompanying the disease: after replacing the mouse *Cx3cr1* locus with the human *CX3CR1^I249/M280^* variant, Cardona et al. observed overt neuronal loss in the cerebellum associated with an exacerbated EAE clinical course, correlating with severe inflammation [121]. On this basis, a role for CX3CR1 in synaptopathy associated with MS might also be speculated. Although conclusive data on synaptopathy are not yet available for the TREM2 and fractalkine/CX3CR1 pathways, taken together these findings suggest a significant involvement of “find and eat me” signalling molecules in MS pathology.

Recent findings show possible interactions between the TREM2 pathway and that of the complement system [122], the most significant “eat me” signal, widely believed to be responsible for early synaptic loss in many neurodegenerative diseases [27,52,123], including MS. The role of the complement in MS pathology is well established, as reviewed by Ingram et al., and it has also been proposed as a biomarker of the disease [124]. Alongside its known role in the opsonization of myelin fragments [124,125,126] and in the progression of the disease [49,127,128], much evidence also suggests its contribution to MS- and EAE-related synaptopathy. In MS, elevated levels of C3 in CSF correlate both with those of the light subunit of the neurofilament protein, marker of ongoing neuronal damage, and with levels of clinical disability [129]. Interestingly, high expression levels of C3 in neural and glial microvesicles extracted from CSF have been found to correlate with low levels of synaptic proteins [130]. The role of C3 in synaptopathy is further supported by evidence showing that a common coding variant of the C3 gene affects not only WM damage but also GM neurodegeneration, and correlates with cognitive impairment [131]. Increased CSF levels of other complement molecules, such as Complement Receptor 2 [132], that are involved in the response to neuronal damage and therefore potentially relevant for immune-mediated synaptopathy, have also been found in MS patients [133].

At the neuropathologic level, while activation of the complement system has been extensively reported in WM demyelinating lesions [125,134,135,136], its role in GM damage is unclear. Low levels of complement deposition were initially observed in purely cortical lesions, together with reduced immunoreactivity for C3d and C4d in combined GM and WM lesions, while enhanced expression of elements of the membrane attack complex (MAC) (C1q, C3d and C5b-9) were found in WM demyelinating lesions [137]. However, in MS, classical (C1q) and alternative (C3b) complement pathway molecules were later detected by immunocytochemistry in cortical neurons located in active lesions, in close proximity to activated microglial cells showing immunoreactivity for CR3, C3aR and C5aR [138]. Activation of the C1q–C3 axis, with deposits of C1q and C3d specifically at the synaptic level and within microglial processes closely related to neurons, has been described in the hippocampus of MS patients. Remarkably, synaptic markers were found to colocalize with microglial processes and lysosomes [68]. A mechanistic contribution in this regard was found in a recent study by Werneburg et al. (2020) [139]. In line with previous findings, they confirmed the occurrence of early synapse loss in the visual thalamus in both MS patients and different EAE models, and highlighted the presence of C3-positive presynaptic terminals selectively engulfed by microglia, but not by astrocytes. In analogy with the temporal profile described for TREM2-dependent aberrant pruning in a model of AD [109], complement-dependent synaptic elimination appeared as an early event in MS and EAE, while microglial-mediated phagocytosis in later stages of the disease seemed essentially to be aimed at clearing myelin debris (Figure 2). Notably, selective C3 inhibition reduced synapse loss and preserved visual circuit functioning, thus suggesting that aberrant pruning in demyelinating diseases is driven preferentially by activation of the alternative complement cascade [139,140], as occurs in other pathologic conditions [141]. This was further confirmed by data showing that, in the chronic progressive EAE model, ablation of C3, but not of C1qa, significantly blunted EAE-induced motor impairment, synapse loss and microglial activation, resulting in an improvement in cognitive performances [142].

In addition to the above, several findings point to the potential involvement of other complement cascade molecules in the pathogenesis of synaptic loss associated with EAE. In particular, antisense-mediated inhibition of peripheral C6, one of the MAC components, has been shown to reduce activation of both the Nod-like receptor protein 3 inflammasome and the MAC complex in the CNS, not only improving the clinical course of the disease, but also reducing the axonal and synaptic damage related to the relapse phase [143].

A contribution to aberrant pruning may also be provided by impairment of molecules related to the “spare me” signalling pathways, in which CD47 and its receptor SIRPα play a prominent role [44]. Indeed, it has been demonstrated that myelin is able to modulate its own phagocytosis by expressing CD47, which, by binding with SIRPα expressed by phagocytes, downregulates this process, thus affording protection from activated microglia [144]. Downregulation of CD47 expression has been reported in WM lesions in MS [145,146,147] and has also been shown to increase phagocytosis of myelin in EAE [147], thus confirming the possibly important contribution of this pathway in modulating the process of clearing myelin debris. However, a specific role in synaptic pruning in MS-associated GM lesions is not yet clear.

Taken together, the findings reported here suggest that the principal player in the complex machinery of aberrant pruning involved in MS-induced synaptopathy is the complement system. However, based on known interactions between the complement system and other signalling pathways, such as TREM2 [122], also deeply involved in MS pathology, the possible contribution of other molecules cannot be excluded and could provide useful avenues for future research. Remarkably, these observations also support the hypothesis that disruptions in the balance between “find me”, “eat me” and “spare me” signals could be responsible for activating aberrant microglial phagocytosis, resulting in excessive synapse loss in the early phase of both MS and experimental models of the disease.

## 4. Conclusions

Early synaptic dysfunction in GM structures involved in cognition is considered the most relevant pathogenic substrate for primitive neuronal damage in MS, leading to progressive CD and seriously affecting the quality of life of patients. In view of the relevance of the emerging contribution of microglia in shaping neuronal wiring in both physiological and pathologic conditions, in this review we have summarized the critical role played by microglia in aberrant synaptic pruning during synapse loss in this disease. As synaptopathy in MS is considered a reversible event [5], an improved understanding of the molecular mechanisms underlying pathological synapse loss, together with identification of the specific time frame during which they damage neurons, will be fundamental to design targeted therapeutic interventions to address CD in this disease by selectively inhibiting the aberrant removal of synapses.

## Data Availability

No new data were created or analyzed in this study. Data sharing is not applicable to this article.
